# Brain Magnetic Resonance Imaging Characteristics of Anti-Leucine-Rich Glioma-Inactivated 1 Encephalitis and Their Clinical Relevance: A Single-Center Study in China

**DOI:** 10.3389/fneur.2020.618109

**Published:** 2021-01-12

**Authors:** Xiali Shao, Siyuan Fan, Huan Luo, Ting Yat Wong, Weihong Zhang, Hongzhi Guan, Anqi Qiu

**Affiliations:** ^1^Department of Radiology, Peking Union Medical College Hospital, Peking Union Medical College and Chinese Academy of Medical Sciences, Beijing, China; ^2^Department of Biomedical Engineering, National University of Singapore, Singapore, Singapore; ^3^Department of Neurology, Peking Union Medical College Hospital, Peking Union Medical College and Chinese Academy of Medical Sciences, Beijing, China; ^4^The N.1 Institute for Health, National University of Singapore, Singapore, Singapore

**Keywords:** MRI, autoimmune diseases, encephalitis, basal ganglia, limbic system

## Abstract

**Objective:** To characterize the magnetic resonance imaging (MRI) features of anti-leucine-rich glioma-inactivated 1 (LGI1) encephalitis and explore their clinical relevance.

**Methods:** Patients with anti-LGI1 encephalitis who underwent MRI at our center were included in this study. Baseline and follow-up MRI characteristics were evaluated, and relationships between lesion location and clinical symptoms were analyzed. The extent of signal abnormalities within the lesion overlap region was measured and correlated with modified Rankin Scale scores and serum antibody titer.

**Results:** Seventy-six patients were enrolled, of which 57 (75%) were classified as MR positive. Brain lesions were located in medial temporal lobe (MTL) (89%) and basal ganglia (BG) (28%). Hippocampus and amygdala were lesion hubs with more than 50% lesion overlap. BG lesions were found in 30% of patients with faciobrachial dystonic seizure (FBDS) and only 7% of patients without FBDS (*p* = 0.013). Meanwhile, MTL lesions were more commonly observed in patients with memory impairment (70 vs. 0%, *p* = 0.017). MRI features included hyperintensity and edema at baseline, as well as hypointensity and atrophy at follow-up. Correlations between signal intensity of lesion hubs (including hippocampus and amygdala) and modified Rankin Scale scores were found on T2 (*r* = 0.414, *p* < 0.001) and diffusion-weighted imaging (*r* = 0.456, *p* < 0.001).

**Conclusion:** MTL and BG are two important structures affected by anti-LGI1 encephalitis, and they are associated with distinctive symptoms. Our study provided evidence from Chinese patients that BG lesions are more commonly observed in patients with FBDS, potentially suggesting BG localization. Furthermore, in addition to supporting diagnosis, MRI has the potential to quantify disease severity.

## Introduction

Anti-leucine-rich glioma-inactivated 1 (LGI1) encephalitis was first reported in 2010 (1, 2). It is the most common autoimmune limbic encephalitis (LE) and the second most common autoimmune encephalitis (AE) after anti-*N*-methyl-d-aspartate receptor (NMDAR) encephalitis (3, 4). The annual incidence of anti-LGI1 encephalitis was reported to be 0.83 per million people in the Dutch population in 2015 (5). Most patients affected by anti-LGI1 encephalitis are men between the ages of 50 and 70 years (3). The clinical manifestations of anti-LGI1 encephalitis include seizure, cognitive impairment, and psychiatric disorder, and one of the most specific symptoms is faciobrachial dystonic seizure (FBDS), which is characterized by frequent, brief, dystonic attacks predominantly involving the face and ipsilateral arm or leg (6, 7). Unlike other types of LE, anti-LGI1 encephalitis is infrequently accompanied by tumors, and typically responds well to immunotherapy (8).

In clinical practice, anti-LGI1 encephalitis has often been misdiagnosed as psychiatric illness or viral encephalitis (9–13), which can delay immunotherapy and cause the deterioration of symptoms. Hyperintensity of the bilateral medial temporal lobe (MTL) on magnetic resonance imaging (MRI) is the typical appearance and diagnostic basis for LE (5, 7, 8, 14, 15). A previous study suggested that cognitive deficits and disability were accompanied by pronounced hippocampal atrophy (8). In addition, accumulating case reports have revealed links between basal ganglia (BG) and FBDS (16–19), suggesting that BG is an important target for this disease. However, the lesion distribution pattern and relationships between lesions and clinical manifestations, especially the association between BG and FBDS, have not been evaluated in large-scale studies. In the current study, we describe the MRI features of patients with anti-LGI1 encephalitis from a single-center retrospective study in China and explore their clinical relevance, in an attempt to increase understanding of the pathogenesis of this disease and provide useful clues for clinical diagnosis and monitoring.

## Methods

### Participants and Study Design

We searched our clinical and radiological database from May 2013 to July 2019. Patients diagnosed with anti-LGI1 encephalitis who underwent brain MRI at our center were included in this study. Patients with significant brain lesions that could not be explained by encephalitis alone were excluded. In this study, patients with symptoms of seizure, cognitive impairment, and/or psychiatric disorder who were positive for anti-LGI1 antibodies in either serum or cerebrospinal fluid were diagnosed with anti-LGI1 encephalitis. The presence of anti-LGI1 antibodies was evaluated using a fixed cell-based indirect immune-fluorescence test using Biochips (Euroimmun AG, Lüebeck, Germany). We retrospectively reviewed patients' clinical data as well as their brain MRI results. Baseline MRI scans were performed on the first outpatient visit or upon admission. Follow-up imaging was acquired from 25 patients at a mean time of 28 months after initial disease onset. Baseline-modified Rankin Scale (mRS) scores were collected as a measurement of disease severity. The scale comprises seven levels of increasingly severe disability, from 0 to 6 (see Supplementary Table 1).

### Image Acquisition

MRI was performed with 3.0-T (Siemens Skyra, GE Discovery, Toshiba Vantage Titan, Philips Ingenia CX) or 1.5-T (GE Signa Excite) scanners with head-neck coils. Most MRI scans incorporated standard brain protocol sequences including fluid-attenuated inversion recovery (FLAIR) [repetition times (TRs) = 7,500–12,000 ms, echo times (TEs) = 81–178 ms, inversion times = 2,000–2,711 ms], T1-weighted imaging (T1WI) (TRs = 1,404–2,309 ms, TEs = 8–29 ms), T2-weighted imaging (T2WI) (TRs = 3,400–13,611 ms, TEs = 82–166 ms), diffusion-weighted imaging (DWI) (TRs = 1,898–6,000 ms, TEs = 62–108 ms), and in some cases contrast-enhanced T1WI (TRs = 1,376–1,946 ms; TEs = 10–29 ms). Slice thicknesses were 4–6.5 mm, and the fields of view were 200–240 mm.

### Image Analysis

Two neuroradiologists, both blinded to patients' clinical details, reviewed the brain MR images to reach consensus. An MR was considered positive when both neuroradiologists identified a signal abnormality or volumetric change in any cortical region or subcortical nuclei; otherwise, the MR was considered negative. MR imaging features were visually assessed including lesion location and signal characteristics. We analyzed the relationship between location and clinical symptoms (memory impairment and FBDS). The time from disease onset to the presence of different MRI characteristics was recorded to explore the evolution of signal features overtime.

**Figure 1:**
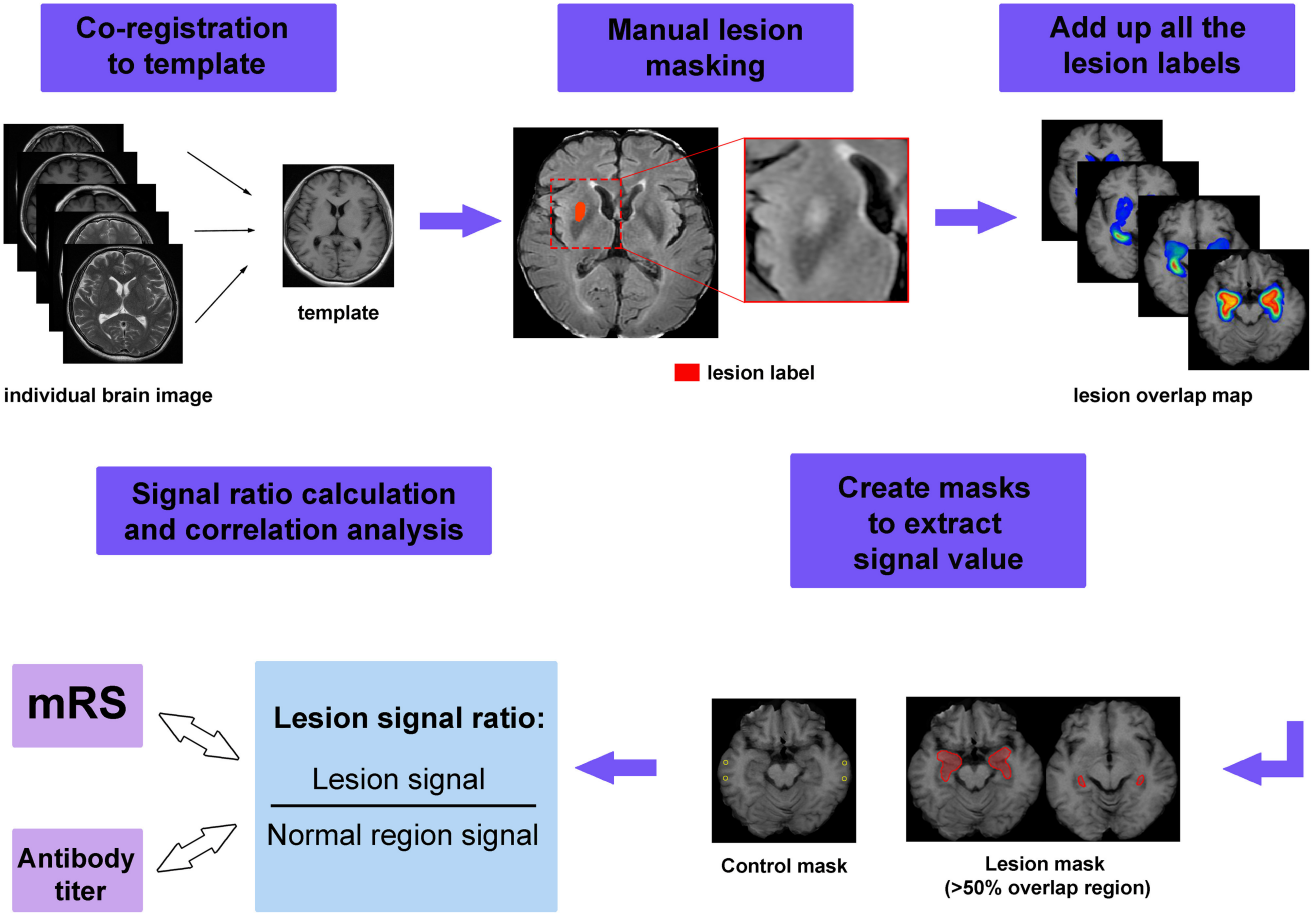
The workflow of image processing. First, the individual image was coregistered to the template and manual lesion masking was performed on the registered image. Next, all lesion labels were added together to form the lesion overlap map. Masks were created to extract the signal value, in which lesion masks were created by applying a threshold of 50% to the overlap map, while control masks were chosen from normal appearing region. Finally, the signal ratios were calculated and used to perform the correlations with modified Rankin Scale (mRS) scores and serum antibody titer.

To show the pattern of lesion distribution, we extracted each patient's lesion and mapped the images onto one brain. This procedure was referred to as lesion overlap mapping. The workflow for image processing is shown in Figure 1. First, the axial images from all the patients were coregistered to a template (one patient's T1 image). Individual lesions were then manually contoured on registered images. Finally, these lesion labels were added together to create an overlap map. To show the difference of lesion extensions between male and female patients, as well as between patients with and without specific symptoms, lesion overlap mapping was also performed separately for these subgroups.

In addition to visual assessment, we also attempted to quantify the extent of signal abnormalities. To achieve this goal, regions of interest (ROIs) were created from the overlap map for all MRI-positive patients to extract signal intensity. We set a threshold for the overlap map and defined areas with more than 50% overlap as lesion ROIs. We aimed to measure the signal intensity in the more commonly affected areas, which could be regarded as lesion hubs for anti-LGI1 encephalitis. To standardize the signal values among different patients, we also measured the signal intensity from tissue that appeared normal. The relative signal was calculated as *S*_ratio_ = *S*_lesion_/*S*_normal_, here *S*_lesion_ and *S*_normal_ denote the average signal intensity of lesion and normal appearing tissue, respectively.

### Statistical Analysis

Statistical analysis was conducted using Statistical Package for the Social Sciences (SPSS) version 22.0 software. The associations between lesion locations and clinical symptoms were explored by χ^2^ test or Fisher's exact test. The differences between the MR-positive and MR-negative group were analyzed using the χ^2^ test or Fisher's exact test for categorical variables and the Mann-Whitney *U* test for continuous variables. Spearman's correlation was used to test the correlation between signal values and mRS/serum antibody titer. False discovery rate was used to adjust for the multiple comparisons. We aimed to explore whether the extent of signal abnormalities was indicative of disease severity. The serum antibody titers were classified into four grades (grade 1 = 1:10, grade 2 = 1:32, grade 3 = 1:100, grade 4 = 1:320), with higher grade indicating higher concentration. A value of *p* < 0.05 was considered to indicate statistical significance.

### Standard Protocol Approvals, Registrations, and Patient Consents

This study was approved by the ethics committee of Peking Union Medical College Hospital (IRB no. JS-891). Written informed consent forms were waived for this retrospective design.

## Results

### Clinical Characteristics

Overall, 76 patients were included in this study. The demographic and clinical characteristics of these patients are listed in Table 1. Of 76 patients, 57 patients (75%) were classified as MR positive and 19 (25%) patients were classified as MR negative. There was no significant difference in clinical features between these two groups.

**Table 1:** Demographic and clinical characteristics of patients with anti-LGI1 encephalitis.

	**Total**	**MRI positive**	**MRI negative**	***p-*value**
**Demographics**				
Age [mean (SD), year]	57.8 (12.8)	57.4 (12.8)	59.1 (12.9)	0.313
Sex (male/female)	52/24	38/19	14/5	0.569
Baseline mRS [mean (SD)]	2 [1]	2 [1]	2 [1]	0.445
Follow-up time [mean (SD), months]^*^	28 [20]	28 [20]	NA	NA
**Serum antibody titer**				
1:10	5 (7%)	4 (7%)	1 (5%)	1
1:32	37 (49%)	28 (49%)	9 (47%)	0.895
1:100	30 (39%)	21 (37%)	9 (47%)	0.5
1:320	4 (5%)	4 (7%)	0	0.569
**Tumor**				
Pituitary adenoma	4 (5%)	3 (4%)	1 (1%)	1
Colorectal adenoma	3 (4%)	2 (3%)	1 (1%)	0.569
Gastric carcinoma	1 (1%)	1 (1%)	0	1
Prostate cancer	1 (1%)	1 (1%)	0	1
Ovarian teratoma	1 (1%)	1 (1%)	0	1
Retroperitoneal ganglioneuroma	1 (1%)	1 (1%)	0	1
Total	11 (15%)	9 (16%)	2 (11%)	0.851
**Symptoms in acute phase**				
Memory impairment	72 (95%)	55 (96%)	17 (89%)	0.553
Seizure	72 (95%)	55 (96%)	17 (89%)	0.553
Faciobrachial dystonic seizure	46 (61%)	35 (61%)	11 (58%)	0.786
Mental and behavior abnormalities	57 (75%)	46 (81%)	11 (58%)	0.092
Sleep disorder	35 (46%)	28 (49%)	7 (37%)	0.352

* *All the follow-up image (the time from initial disease onset to the last follow-up) was only available in MRI-positive patients*.

*NA, not applicable*.

### Lesion Locations

**Figure 2:**
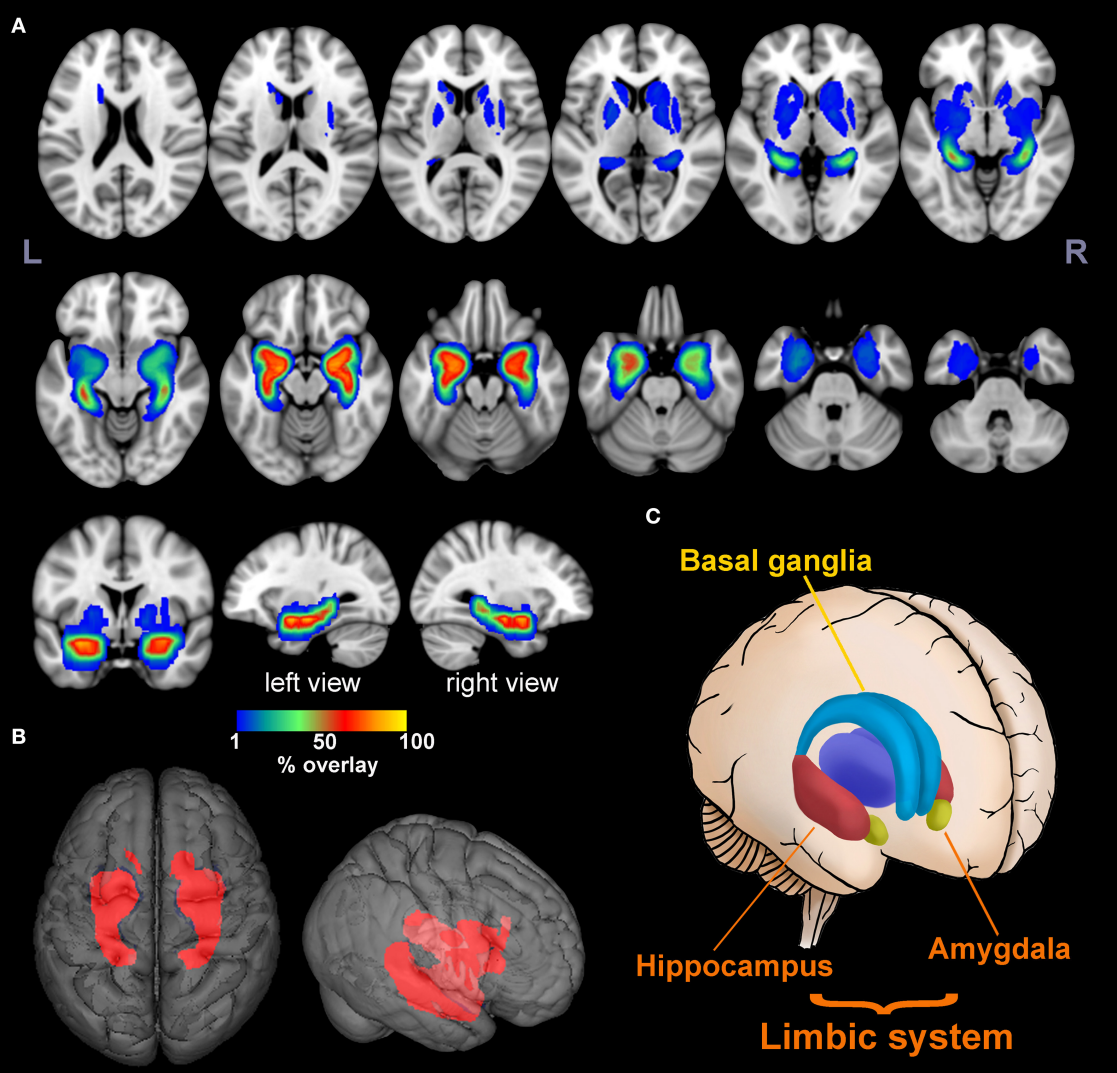
Lesion overlap map. The multislice view **(A)** and 3D view **(B)** of the lesion overlap map for 57 MR-positive patients. The color bar indicates the percentage of lesion overlay. The brain regions involved mainly included the limbic system and basal ganglia as illustrated in **(C)**. More than 50% of the lesions overlapped in the hippocampus and amygdala [hot colors in **(A)**].

The lesion overlap map showed that, in addition to classic limbic system involvement, BG was another important target for anti-LGI1 encephalitis (Figure 2). Specifically, 51 lesions (89%) were found to be located in MTL, while 16 lesions (28%) were located in BG. More than 50% of the lesions overlapped at bilateral hippocampus and amygdala, which could be regarded as lesion hubs. The overlap maps for male and female patients were similar (Supplementary Figure 1).

### Lesion Signal Characteristics

**Figure 3:**
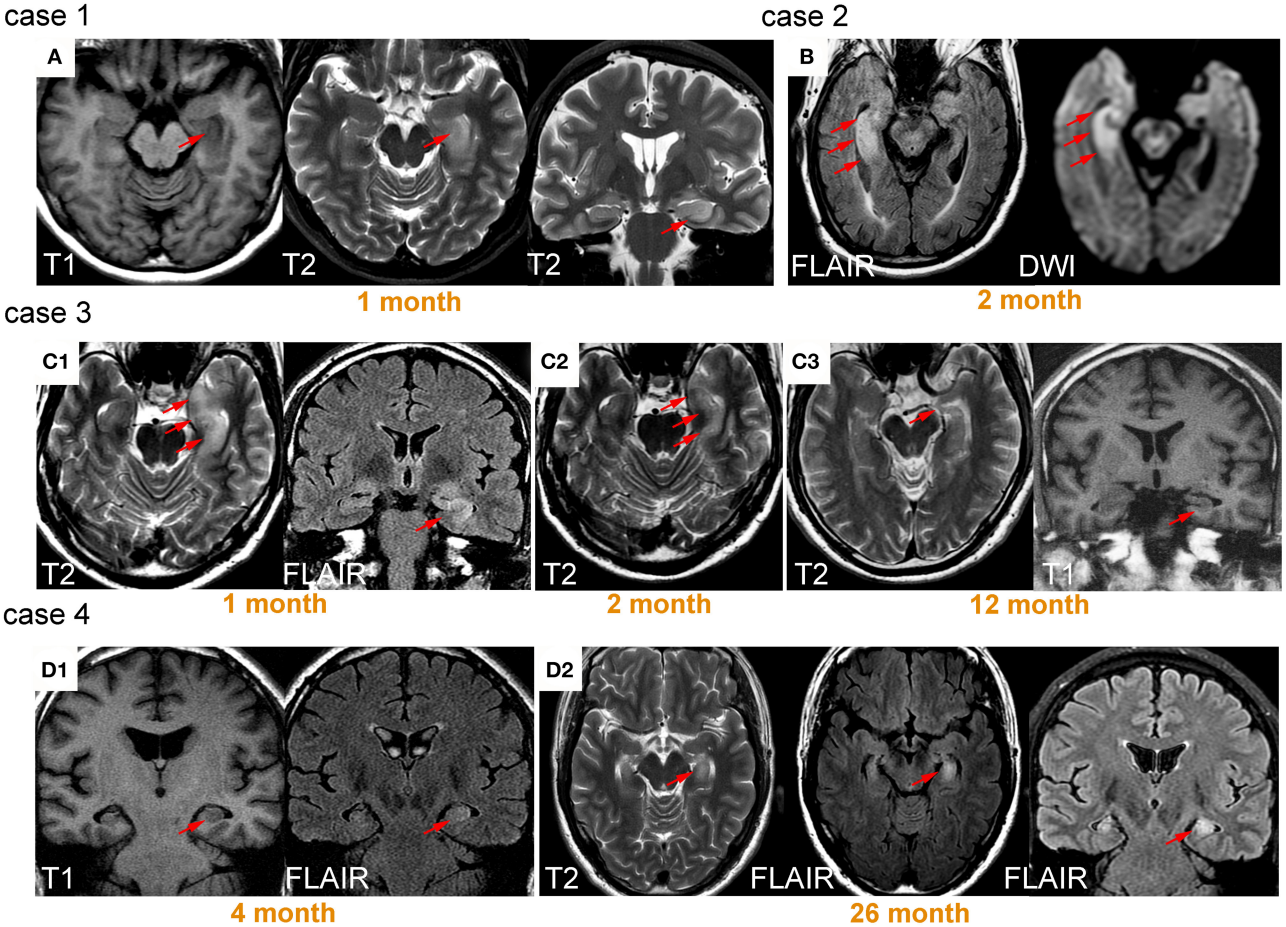
Lesions on medial temporal lobe and their signal change over time. Case 1 was a 59-year-old female patient presenting with memory impairment and seizure. T1 hypointensity and T2 hyperintensity accompanied by swelling were observed on left medial temporal lobe (MTL) 1 month after onset **(A)**. Case 2 was a 73-year-old male patient who presented with seizure for 2 months, exhibiting prominent right MTL swelling and hyperintensity **(B)**. Case 3 was a 52-year-old male patient with seizure. Hyperintensity and swelling in left MTL was observed 1 month after onset **(C1)**. After treatment with dexamethasone, the patient's symptoms improved, and the area of signal abnormality decreased **(C2)**. Twelve months after onset, prominent atrophy occurred **(C3)**. The patient did not fully recover from memory deficits. Case 4 was a 43-year-old male patient with seizure and memory impairment. Slight swelling and hyperintensity were observed in left MTL **(D1)**. The patient's symptoms were not well controlled even after immunotherapy. Swelling and hyperintensity became more prominent 26 months later **(D2)**.

Because brain lesions can be classified into two types according to the involvement of the MTL or BG, the signal patterns were evaluated separately. For MTL lesions, images usually presented as hypointensity on T1WI, and hyperintensity on T2WI, FLAIR, and DWI, accompanied by edema. Atrophy was usually observed in the late stage. Of 21 patients with follow-up imaging, brain MRI normalized in three patients (14%), nine patients (43%) developed atrophy, and 11 patients (52%) exhibited persistent high signal without loss of volume. Illustrative cases are shown in Figure 3.

**Figure 4:**
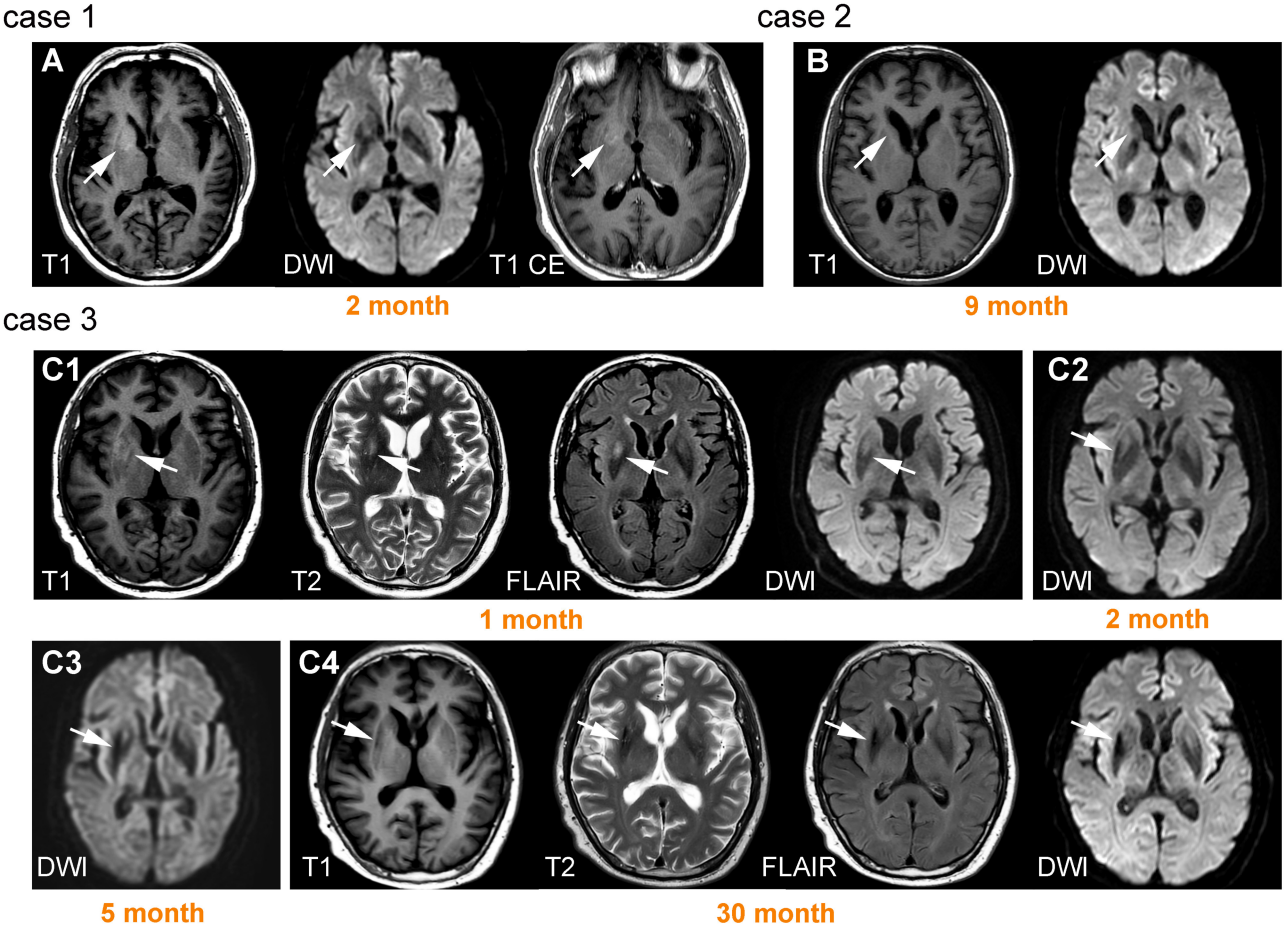
Lesions on basal ganglia and their signal change over time. Case 1 was a 66-year-old male patient with left faciobrachial dystonic seizure (FBDS). A lesion was observed on the right basal ganglia (BG) with T1 hyperintensity and diffusion-weighted imaging (DWI) hypointensity, accompanied by ring-shaped enhancement **(A)**. Case 2 was a 57-year-old male patient with epileptic seizure. Brain MRI showed prominent right caudate nucleus atrophy and DWI hypointensity 9 months after onset **(B)**. Case 3 was a 61-year-old male patient presenting with left FBDS. Hyperintensity on T1, T2, fluid-attenuated inversion recovery (FLAIR), and DWI were initially observed on right BG **(C1)**. Hypointensity on DWI began to appear 2 months after onset **(C2)** and became more prominent accompanied by atrophy 3 months later **(C3)**. High signals were replaced by hypointensity 30 months after onset **(C4)**.

The signal patterns of BG lesions were more complex and could be mixed combinations of hypo- or hyperintensity on T1WI, T2WI, DWI, and FLAIR. However, it appeared that hyperintensity (either on T1WI, T2WI, DWI, and FLAIR) usually only occurred in the early stage with a median time interval of 1–3 months after onset. In contrast, hypointensity (either on T1WI, T2WI, DWI, and FLAIR) and atrophy usually presented in the chronic phase. Follow-up images were available in six patients. Of these, four patients (67%) exhibited persistent signal abnormalities without volume loss, and two patients (33%) developed atrophy. Illustrative cases are shown in Figure 4.

### The Relationship Between Lesion Location and Clinical Symptoms

To explore the relationship between lesion location and symptoms, we compared the lesion presentation frequency between patients with or without specific symptoms. As a result, BG lesions were more likely to be found in patients with FBDS (30%) compared with those without FBDS (7%) (*p* = 0.013). In addition, BG lesions were less common in patients with memory impairment (18%) compared with those without memory impairment (75%) (*p* = 0.037). MTL lesions were found in 70% of patients with memory impairment but were not found in any patients without memory impairment (*p* = 0.017). A comparison of lesion overlap maps for patients with and without specific symptoms could be found in Supplementary Figure 2.

### Correlation Between Signal Measurements and Disease Severity

**Figure 5:**
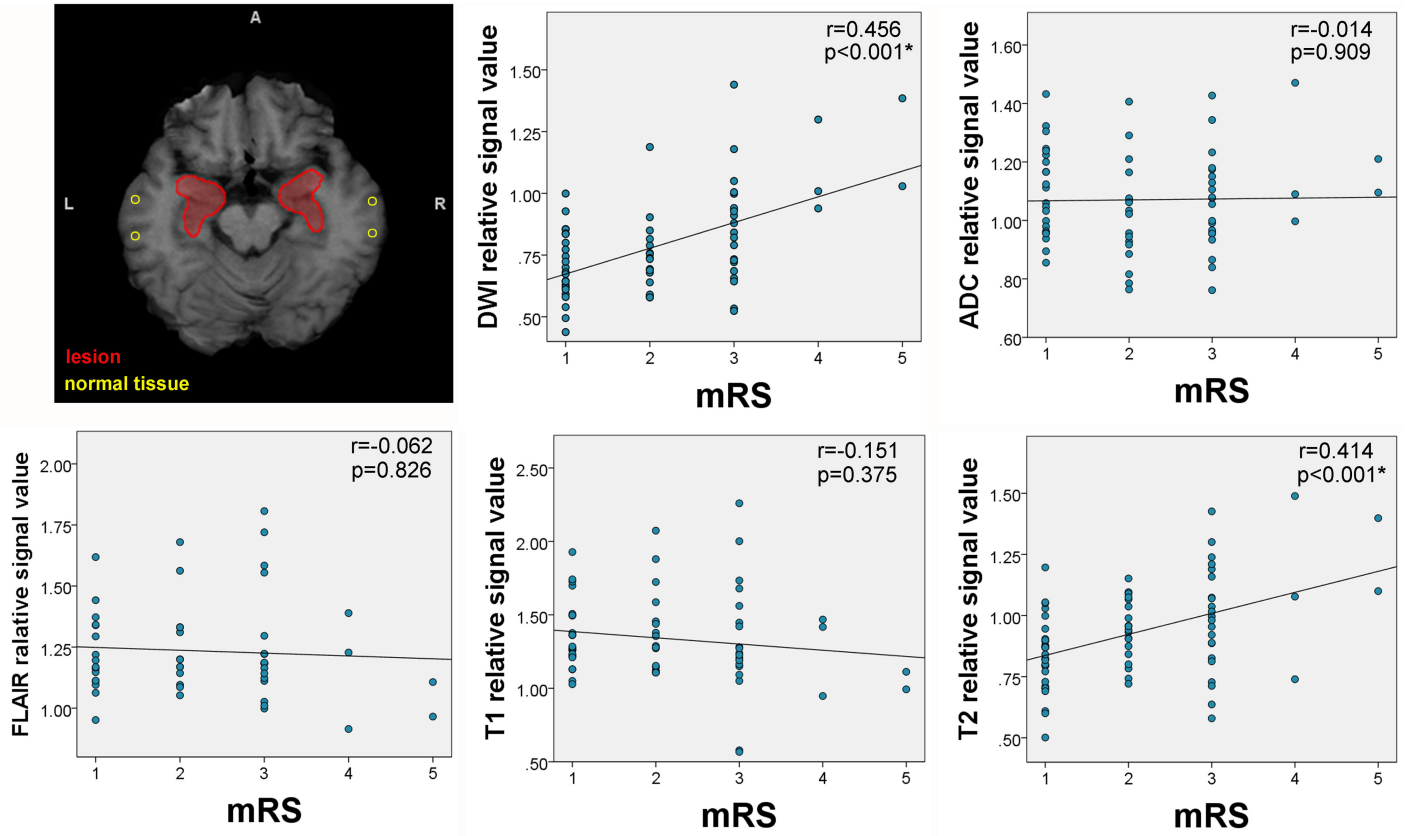
Correlation between mRS and lesion signal measurements. The upper left corner shows the placement of regions of interest (ROIs) for signal measurements. The areas circled by red lines were created from lesion overlap maps and were used to extract lesion signals. The circular ROIs were used to extract normal tissue signals. Correlations between signal intensity and modified Rankin Scale (mRS) scores were observed on T2 and diffusion-weighted imaging (DWI).

The quantitative signal measurements were performed on T1WI, T2WI, FLAIR, DWI, and apparent diffusion coefficient (ADC) for the lesion hubs, then correlated with mRS scores and serum antibody titer, respectively. The results revealed correlations between relative signal intensity and mRS scores on T2WI (*r* = 0.414, *p* < 0.001) and DWI (*r* = 0.456, *p* < 0.001) (see Figure 5). However, no correlation was found between serum antibody titers and signal values for any sequence.

## Discussion

The current study investigated brain MRI results of 76 patients with anti-LGI1 encephalitis and explored their clinical relevance. To the best of our knowledge, this is the largest study providing detailed descriptions of the MRI-based characteristics of Chinese patients. Moreover, in addition to visual assessment, we also tried to find disease-specific biomarkers through quantitative analysis. There are several major findings: first, brain lesions of patients with anti-LGI1 encephalitis were mainly located in MTL and BG as has been previously described (15, 18, 20–22); second, MTL lesions were more likely to be observed in patients with memory impairment, whereas BG lesions were more commonly observed in patients with FBDS; third, the extent of signal abnormalities in the lesion area was correlated with disease severity, as measured by mRS scores.

Anti-LGI encephalitis exhibited classic limbic system involvement with its core hubs located in the hippocampus and amygdala. This finding is in accord with previous pathophysiological findings showing that the LGI1 protein is mainly expressed in the hippocampal region and temporal cortex (23). Correspondingly, most patients in the current study presented with memory impairment and seizure (95% for both conditions). A significant correlation between MTL lesions and memory impairment was demonstrated, suggesting MTL to be responsible for memory deficits. This observation is also consistent with previous studies reporting the linking between hippocampal atrophy and memory impairment in these patients (24, 25).

More importantly, we have observed a correlation of BG lesions with FBDS. This finding may contribute to the understanding of the pathogenesis of FBDS which remains extensively debated. Increasing evidence tends to suggest basal ganglia localization for FBDS, because structural and functional changes in BG are more easily detected in subjects with FBDS (18, 21, 22). However, cohort studies in Germany and Netherlands failed to find such association (3, 15). This controversy may be related to ethnic differences or varied disease courses among different cohorts. In any case, the current study provided evidence from a Chinese cohort which further supports the notion that basal ganglia are involved in the development of FBDS. FBDS typically precedes the onset of anti-LGI1 encephalitis (7). Early treatment can prevent progression to LE and development of cognitive deficits (26, 27). Therefore, when evaluating a movement disorder suggestive of FBDS, the finding of BG lesions should reinforce the suspicion of anti-LGI1 encephalitis.

Anti-LGI encephalitis can mimic a variety of other pathological processes because of its diverse clinical features, presenting a diagnostic challenge for clinicians. This condition is commonly misdiagnosed as Creutzfeldt-Jakob disease and viral encephalitis (9, 28–31). Thus, the recognition of lesion distribution pattern could serve as a useful clue for differential diagnosis. Unlike anti-LGI1 encephalitis, which is usually restricted to MTL, viral encephalitis may develop more extensive MRI abnormalities in frontal, occipital, or parietal lobes (29, 32). In addition, even though both viral encephalitis and LE affect the MTL, BG involvement favors nonviral etiologies (32).

The signal pattern of MTL lesions was typical for LE, which usually manifests as T2-FLAIR hyperintensity in the acute phase and mesial temporal sclerosis on follow-up imaging (14). The current results are in accord with these general findings and hyperintensity and edema were both prominent in the acute phase. Follow-up MRI revealed normalization in only 13% of patients, with most patients exhibiting either persistent high signal (52%) or developed atrophy (43%). Long-lasting hyperintensity and atrophy may have been associated with persistent neurological deficits as illustrated in our cases (cases 3 and 4 in Figure 3). The BG signal pattern has not been fully described before, with only a few studies reporting T1 hyperintensity in FBDS patients (16–19, 33). In the current study, the results revealed that mixed signal abnormalities with different sequences could be observed in BG. Overall, hyperintensity only presented in the early stage, whereas hypointensity and atrophy were usually observed in the chronic phase. Unlike a previous study (18), we did not observe resolution of abnormal BG signals at follow-up. Instead, these signals developed into hypointensity and atrophy, potentially related to immune-mediated brain injury.

Furthermore, the current findings have demonstrated that the extent of signal abnormality in lesion hubs was able to reflect disease severity. Specifically, signal values of T2 and DWI were positively correlated with mRS scores. Interestingly, decreased ADC values was not observed in cases with increased DWI. It appears that elevated DWI may have reflected the T2 “shine-through” effect but not restricted diffusion, as commonly seen in cases of abscess (34). Thus, elevated T2 and DWI may have reflected the underlying interstitial edema caused by inflammation but not cytotoxic damage, and the extent of edema is indicative of disease severity. However, it should be noted that the strength of correlation coefficients was only moderate. This may probably be because we did not take volumetric changes into consideration which are also reported to be important for reflecting neurological deficits (15, 26). Because of the retrospective nature of the current study, we were unable to perform volumetric analysis. Nonetheless, we demonstrated that it is feasible to use routine clinical scan to evaluate and quantify the extent of damage.

The current study involved several limitations that should be considered. First, the imaging findings were likely to have reflected not only the primary pathological autoimmune inflammatory process but also alterations associated with immunotherapy and antiepileptic drug treatments. However, we were unable to disentangle these factors because of variation in disease duration and medical treatments among different patients. Second, because of the retrospective nature of the study, brain MRI was not regularly performed for all patients at follow-up and more advanced techniques, such as high-resolution MRI were not performed for calculating the exact volume of important structures. Advanced diffusion techniques such as diffusion tensor imaging (35), neurite orientation dispersion and density imaging (36), and diffusion kurtosis imaging (37, 38) should be implemented in future studies to investigate microstructural changes caused by anti-LGI1 encephalitis. Third, because a moderate proportion of patients showed no brain lesions, functional MRI and electroencephalography should be conducted to further assess the underlying functional changes. Finally, clinical measurement only included mRS. The main criticism of this method is that the grades are too broad and ill-defined (39), lacking sufficient sensitivity to reflect small differences between individuals. A more systematic assessment including disease-specific neurocognitive evaluation should be performed in future studies.

## Conclusion

In conclusion, the present study reviewed brain MRI results of patients with anti-LGI1 encephalitis and identified several characteristic radiological patterns. Classic limbic system involvement and the typical signal features on MTL supported the diagnosis of LE. BG was another important structure in the pathogenesis of anti-LGI1 encephalitis, which may be involved in the development of FBDS. Furthermore, MRI showed the potential to reflect disease severity, suggesting that it may be a useful biomarker for tracking disease-related changes.

## Data Availability Statement

The original contributions generated for the study are included in the article/Supplementary Material, further inquiries can be directed to the corresponding author/s.

## Ethics Statement

The studies involving human participants were reviewed and approved by the ethics committee of Peking Union Medical College Hospital (IRB no. JS-891). Written informed consent for participation was not required for this study in accordance with the national legislation and the institutional requirements.

## Author Contributions

HG, WZ, and AQ contributed to the design of the study. XS, SF, TW, and HL performed the analysis. XS and SF wrote the first draft of the manuscript. All authors contributed to the article and approved the submitted version.

## Conflict of Interest

The authors declare that the research was conducted in the absence of any commercial or financial relationships that could be construed as a potential conflict of interest.

## Supplementary Material

Supplementary Figure 1 Lesion overlap maps for male and female patients. The lesion overlap maps were very similar of male and female patients, except for some differences in the location of basal ganglia lesions.

Supplementary Figure 2 Lesion overlap maps for patients with and without faciobrachial dystonic seizure. There were more basal ganglia lesions in patients with faciobrachial dystonic seizure (FBDS) compared with those without FBDS.

Supplementary Table 1 Modified Rankin Scale for neurologic disability.
