# Supplementary material for: Brain Magnetic Resonance Imaging Characteristics of Anti-Leucine-Rich Glioma-Inactivated 1 Encephalitis and Their Clinical Relevance: A Single-Center Study in China
Source: Front Neurol. 2021 Jan 12;11:618109. doi: 10.3389/fneur.2020.618109 (PMC7835512; doi:10.3389/fneur.2020.618109)
Supplement: Supplementary Table 1 — Modified Rankin Scale for neurologic disability. [file Table_1.DOCX]

**Table S1. Modified Rankin Scale for neurologic disability.**

| **Score** | **Description** |
| --- | --- |
| 0 | No symptoms at all |
| 1 | No significant disability despite symptoms; able to carry out all usual duties and activities |
| 2 | Slight disability; unable to carry out all previous activities, but able to look after own affairs without assistance |
| 3 | Moderate disability; requiring some help, but able to walk without assistance |
| 4 | Moderately severe disability; unable to walk without assistance and unable to attend to own bodily needs without assistance |
| 5 | Severe disability; bedridden, incontinent, and requiring constant nursing care and attention |
| 6 | Dead |
